# Natural Ellagitannins as Potential Therapeutic Agents for Metabolic Syndrome-Related Disorders

**DOI:** 10.3390/foods15173114

**Published:** 2026-09-02

**Authors:** Chika I. Chukwuma, Nomonde P. Mapasa, Lifted T. Olusola, Lesego P. Kgasapane

**Affiliations:** 1 Centre for Quality of Health and Living (CQHL), Faculty of Health and Environmental Sciences, Central University of Technology, Bloemfontein 9301, Free State, South Africa; 224107123@stud.cut.ac.za (N.P.M.); 223095708@stud.cut.ac.za (L.T.O.); 218012313@cutfs.onmicrosoft.com (L.P.K.); 2 Department of Health Sciences, Faculty of Health and Environmental Sciences, Central University of Technology, Bloemfontein 9301, Free State, South Africa

**Keywords:** ellagitannins, pharmacological potential, metabolic syndrome, insulin signaling, dyslipidemia, hypertension

## Abstract

Over the years, metabolic syndrome (MetS) has emerged as one of the most significant public health challenges, driven by the global increase in obesity, physical inactivity, unhealthy dietary patterns, and population aging. Ellagitannins are a class of hydrolysable tannins that have emerged as promising natural bioactive compounds, owing to their broad spectrum of biological activities. Increasing evidence suggests that some ellagitannins and their metabolites modulate several pathogenic mechanisms underlying metabolic syndrome, including oxidative stress, chronic inflammation, insulin resistance, endothelial dysfunction, and dysregulated lipid metabolism. This work reviews the pharmacological activities and therapeutic prospects of ellagitannins in the management of metabolic syndrome-related disorders. It further provides a critical and comparative analysis of the pharmacological profiles of reported ellagitannins, offering insights into their therapeutic efficacy and potential synergistic applications in the management of MetS-related disorders. Overall, Punicalagin and Geraniin currently possess the strongest preclinical evidence, supported by multiple independent studies employing both cellular and animal models that consistently demonstrate beneficial effects across several components of MetS. Chebulagic acid, Corilagin, Chebulinic acid, and Punicalin also show considerable promise but require further validation in diverse experimental systems. Potential synergistic interactions among ellagitannins when administered in combination should be investigated for enhanced therapeutic efficacy and broader multifaceted pharmacological effects.

## 1. Overview of Metabolic Syndrome

Metabolic syndrome (MetS) is a multifactorial metabolic disorder characterized by the coexistence of interconnected cardiometabolic abnormalities, including central obesity, hypertension, dyslipidemia, hyperglycemia, and insulin resistance. Collectively, these abnormalities substantially increase the risk of developing type 2 diabetes mellitus (T2D), cardiovascular disease (CVD), and other chronic non-communicable diseases [1]. Rather than representing a single disease entity, MetS encompasses a cluster of interrelated metabolic disturbances driven by common pathophysiological mechanisms, including insulin resistance, chronic low-grade inflammation, oxidative stress, adipose tissue dysfunction, and endothelial impairment [2].

Several organizations have proposed diagnostic criteria for MetS, including the World Health Organization (WHO), the National Cholesterol Education Program Adult Treatment Panel III, the International Diabetes Federation, and the Joint Interim Statement (JIS) [2,3,4]. Among these, the JIS criteria are widely used in both clinical practice and epidemiological studies. A diagnosis of MetS is established when an individual presents with any three or more of the following five components [5]:

1. Elevated waist circumference, with cut-off values defined according to population- and country-specific criteria (e.g., ≥102 cm for European men and ≥88 cm for European women);
2. Elevated fasting triglycerides (TG) (≥150 mg/dL [1.7 mmol/L]) or receiving treatment for elevated triglycerides;
3. Reduced high-density lipoprotein cholesterol (HDL-C) (<40 mg/dL [1.03 mmol/L] in men and <50 mg/dL [1.29 mmol/L] in women) or receiving treatment for reduced HDL-C;
4. Elevated blood pressure (systolic ≥130 mmHg and/or diastolic ≥85 mmHg) or current antihypertensive treatment;
5. Elevated fasting plasma glucose (≥100 mg/dL [5.6 mmol/L]) or previously diagnosed type 2 diabetes.

The diagnostic thresholds vary slightly among different international organizations, but all definitions emphasize the coexistence of multiple metabolic abnormalities that synergistically increase cardiometabolic risk [6]. These diagnostic criteria facilitate early identification and management of MetS in high-risk individuals, supporting timely implementation of lifestyle modifications and pharmacological interventions aimed at preventing disease progression and reducing the burden of cardiometabolic complications [3].

Over the years, MetS has emerged as one of the most significant public health challenges, driven by the global increase in obesity, physical inactivity, unhealthy dietary patterns, and population aging [4]. Current estimates indicate that MetS affects approximately 25–30% of the global adult population, with a pooled prevalence of approximately 32.4% reported in Africa. Epidemiological studies further suggest that the prevalence of MetS is generally higher among women than men in many populations, although this varies according to geographic region, ethnicity, and the diagnostic criteria used [3]. The prevalence of MetS continues to rise in parallel with increasing rates of obesity and other lifestyle-related cardiometabolic risk factors.

## 2. Metabolic Syndrome-Related Disorders

The development and progression of metabolic syndrome are closely associated with several metabolic disorders, with insulin resistance and visceral obesity being recognized as the primary pathogenic drivers [7]. Insulin resistance is defined as a diminished biological response of insulin-sensitive tissues, including skeletal muscle, adipose tissue, and the liver, to the actions of circulating insulin. Consequently, higher concentrations of insulin are required to maintain glucose homeostasis, leading to compensatory hyperinsulinemia and, ultimately, impaired glucose regulation and the development of T2D [8]. Beyond glucose dysregulation, insulin resistance contributes to dyslipidemia, hypertension, endothelial dysfunction, and chronic low-grade inflammation, thereby serving as a central mechanism linking MetS to its associated cardiometabolic complications [9].

### 2.1. Type 2 Diabetes Mellitus

T2D is one of the most prevalent and clinically significant disorders associated with MetS. It is a chronic metabolic disease characterized by persistent hyperglycemia resulting from a combination of insulin resistance and progressive pancreatic β-cell dysfunction [8]. The close relationship between T2D and MetS arises from their shared pathophysiological mechanisms, including visceral obesity, chronic low-grade inflammation, oxidative stress, and impaired insulin signaling [1]. Prolonged insulin resistance increases the demand for insulin secretion, ultimately leading to β-cell exhaustion and impaired glucose homeostasis. Consequently, individuals with MetS are at a substantially increased risk of developing T2D and its associated complications, including nephropathy, retinopathy, neuropathy, and CVD [3].

### 2.2. Obesity

Similarly, obesity, particularly visceral or central obesity, plays a pivotal role in the pathogenesis of MetS. The WHO defines obesity as abnormal or excessive fat accumulation that presents a risk to health and classifies it as a chronic, multifactorial disease influenced by genetic, environmental, and behavioral factors. Clinically, obesity is commonly defined by a body mass index of ≥30 kg/m^2^ [8]. Excess adipose tissue, particularly visceral fat, functions as an active endocrine organ that secretes adipokines, pro-inflammatory cytokines, and free fatty acids, thereby promoting insulin resistance, oxidative stress, and systemic inflammation. Consequently, T2D and MetS share several common pathophysiological mechanisms, including insulin resistance and metabolic disturbances arising from adipose tissue dysfunction [1].

### 2.3. Dyslipidemia

Dyslipidemia, a major component of MetS, is characterized by elevated plasma triglyceride concentrations, reduced HDL-C, and, in some cases, increased levels of low-density lipoprotein cholesterol (LDL-C) [3]. These lipid abnormalities promote the development of atherosclerosis, thereby substantially increasing the risk of cardiovascular events, including myocardial infarction and stroke [10]. The principal mechanism underlying dyslipidemia in MetS is insulin resistance. Under normal physiological conditions, insulin suppresses lipolysis in adipose tissue. However, insulin resistance impairs this regulatory effect, resulting in increased lipolysis and excessive release of free fatty acids into the circulation [7]. The increased flux of free fatty acids to the liver stimulates hepatic triglyceride synthesis and very-low-density lipoprotein production, further exacerbating dyslipidemia and contributing to the progression of CVD [3].

### 2.4. Hypertension

Hypertension is another major component of MetS and is largely driven by the interplay between insulin resistance and compensatory hyperinsulinemia. These conditions stimulate sympathetic nervous system activity and activate the renin–angiotensin–aldosterone system (RAAS), thereby contributing to increased vascular resistance and elevated blood pressure [11]. Furthermore, insulin resistance promotes renal sodium reabsorption and impairs endothelial nitric oxide production, resulting in reduced vasodilation and further exacerbation of hypertension. This complex pathophysiological process is further amplified by adipose tissue dysfunction, particularly in visceral obesity, where adipocytes synthesize and secrete angiotensinogen, the precursor of angiotensin II [8]. Increased production of adipose-derived angiotensinogen contributes to chronic activation of the RAAS, establishing a critical mechanistic link between obesity, insulin resistance, and hypertension in MetS [3]. Persistent activation of these pathways ultimately promotes endothelial dysfunction, vascular remodeling, and an increased risk of cardiovascular complications [7].

### 2.5. Metabolic Dysfunction-Associated Steatotic Liver Disease (MASLD)

Metabolic dysfunction-associated steatotic liver disease (MASLD), formerly known as non-alcoholic fatty liver disease (NAFLD), comprises a spectrum of liver disorders characterized by excessive accumulation of triglycerides within hepatocytes in the absence of significant alcohol consumption [12]. MASLD is considered the hepatic manifestation of MetS and is strongly associated with central obesity, insulin resistance, dyslipidemia, and chronic low-grade inflammation. Increased flux of free fatty acids from adipose tissue to the liver, coupled with enhanced de novo lipogenesis and impaired lipid oxidation, promotes hepatic steatosis and may ultimately progress to steatohepatitis, fibrosis, cirrhosis, and hepatocellular carcinoma [8].

### 2.6. Cardiovascular Diseases

CVD is an umbrella term encompassing a group of disorders affecting the heart and blood vessels and remains the leading cause of morbidity and mortality associated with MetS. The majority of cardiovascular events arise from atherosclerosis, a chronic inflammatory condition characterized by the formation of lipid-rich plaques within the arterial wall [9]. The coexistence of insulin resistance, dyslipidemia, hypertension, obesity, and systemic inflammation accelerates atherogenesis and substantially increases the risk of adverse cardiovascular outcomes, including coronary artery disease, myocardial infarction, heart failure, and stroke [8].

## 3. Current Therapeutic Approaches and Limitations

Current therapeutic approaches for MetS primarily focus on managing its individual components and reducing overall cardiometabolic risk through lifestyle modification, pharmacotherapy, and, in selected cases, surgical interventions [3].

### 3.1. Lifestyle Interventions

Lifestyle interventions remain the cornerstone and first-line strategy for the prevention and management of MetS, with particular emphasis on dietary modification, increased physical activity, weight reduction, and behavioral changes. Numerous studies have demonstrated that improvements in lifestyle factors can significantly reduce insulin resistance, improve lipid profiles, lower blood pressure, and decrease the risk of progression to T2D and CVD [7].

Dietary modification plays a crucial role in the management of MetS. A balanced diet rich in fruits, vegetables, whole grains, legumes, and unsaturated fats has been associated with significant improvements in metabolic health indicators. Plant-based diets have an abundance of bioactive compounds possessing antioxidant and anti-inflammatory properties, which contribute to improved insulin sensitivity, reduced oxidative stress, and attenuation of chronic inflammation [10].

Physical activity plays a fundamental role in the prevention and management of MetS by reducing adiposity, improving insulin sensitivity, and enhancing overall metabolic health [3]. Exercise induces metabolic and mitochondrial adaptations in skeletal muscle, thereby increasing insulin-dependent glucose uptake and improving hyperinsulinemia [13]. Furthermore, exercise promotes inter-organ communication through the secretion of myokines, which exert beneficial effects on glucose and lipid metabolism [9]. It also promotes mitochondrial biogenesis and activates several metabolic signaling pathways, including adenosine monophosphate-activated protein kinase, leading to enhanced energy metabolism and increased glucose uptake by skeletal muscle [3]. Additionally, regular exercise improves glycogen storage, contributes to blood glucose regulation, lowers blood pressure, and reduces the risk of cardiovascular disease [3].

### 3.2. Pharmacotherapy

For individuals with T2D, impaired glucose tolerance, and persistent hyperglycemia, commercial blood glucose-lowering agents are usually the clinically recommended option to manage T2D-related metabolic disorders. The blood glucose-lowering agents include the glucagon-like peptide-1 (GLP-1) receptor agonists, biguanides, thiazolidinediones, sulfonylureas, sodium–glucose co-transporter-2 (SGLT-2) inhibitors, meglitinides, dipeptidyl peptidase-4 (DPP-4) inhibitors, and alpha-glucosidase inhibitors [14].

Additionally, in the presence of obesity and cardiovascular diseases, other commercially available drugs, such as antihypertensive drugs (angiotensin-converting enzyme inhibitors, angiotensin receptor blockers, diuretics, angiotensin receptor–neprilysin inhibitors, β-lockers, etc.), cardioprotective drugs (statins, antiplatelet agents, anticoagulants, antiarrhythmics, etc.), and anti-obesogenic agents may be used in combination with blood glucose-lowering agents [15,16].

Some approaches have targeted inflammatory signaling. It is believed that drugs targeting inflammatory pathways, such as nuclear factor kappa B and c-Jun-N-terminal kinase pathways, could potentiate beneficial effects on metabolic syndrome [17,18]. Other experts are of the opinion that immune cell regulation could be another therapeutic target. Specifically, it is believed that reducing the infiltration of immune cells into adipocytes may be of benefit in managing metabolic syndrome [19]. Furthermore, the role of antioxidant therapies cannot be overemphasized. It is well-known that oxidative stress is implicated in the pathophysiology of metabolic syndrome and the associated pro-inflammatory disorders [20]. Hence, antioxidant supplements can help to improve mitochondrial function and reduce oxidative stress-mediated inflammation in metabolic syndrome [17,21].

### 3.3. Surgical Interventions

Surgical interventions are generally considered when lifestyle modifications and pharmacological therapies fail to achieve therapeutic goals or when immediate intervention is required because of severe obesity and associated comorbidities [7]. Bariatric surgery is widely regarded as the most effective therapeutic option for achieving substantial and sustained weight loss and for improving obesity-related comorbidities, particularly T2D [22]. Procedures such as sleeve gastrectomy and Roux-en-Y gastric bypass have demonstrated remarkable efficacy in inducing long-term weight reduction and promoting the remission of T2D, hypertension, and dyslipidemia. These beneficial effects are mediated not only by caloric restriction and malabsorption but also by profound alterations in gut hormone secretion, bile acid metabolism, and the composition of the gut microbiota [23].

Most times, synthetic medications have side effects, while lifestyle modifications may fail to achieve therapeutic targets. Hence the need for novel therapeutic strategies capable of simultaneously targeting the multiple pathophysiological mechanisms underlying metabolic syndrome [24]. Consequently, there is increasing interest in naturally derived bioactive compounds, particularly polyphenols, as alternative or complementary therapeutic agents.

## 4. Natural Products as Alternative Therapeutics in Metabolic Disease Management

To date, the potential of natural products in the prevention and management of MetS has been extensively investigated because of their diverse biological activities and favorable safety profiles [25]. Natural products, particularly those derived from plants, are rich sources of bioactive compounds with antioxidant, anti-inflammatory, and metabolic-modulating properties that may target multiple pathways involved in the pathogenesis of MetS [26]. Numerous medicinal plants and plant-derived preparations have demonstrated beneficial effects on key components of MetS, including hyperglycemia, dyslipidemia, obesity, hypertension, and abnormal cholesterol and triglyceride levels [27].

Among these bioactive constituents, polyphenols have attracted considerable attention because of their ability to modulate oxidative stress, inflammation, glucose metabolism, and lipid homeostasis. Plant-derived polyphenols and their metabolites have long been recognized for their potent antioxidant properties and relatively low incidence of adverse effects compared with many conventional pharmacological agents [28]. Consequently, increasing research efforts have focused on identifying specific classes of polyphenols with multitarget therapeutic potential for MetS. Among these, ellagitannins, a group of hydrolysable tannins found in fruits, nuts, and medicinal plants, have emerged as particularly promising candidates owing to their antioxidant, anti-inflammatory, antidiabetic, antihypertensive, and cardioprotective activities [29].

## 5. Ellagitannins as Emerging Therapeutic Candidates

### 5.1. Rationale, Scope, Conceptual Framework, and Strategy of Review

Ellagitannins are a class of hydrolysable tannins that have emerged as promising natural bioactive compounds owing to their broad spectrum of biological activities. Upon hydrolysis, they release ellagic acid, which is subsequently metabolized by the gut microbiota into bioactive urolithins. Increasing evidence suggests that these metabolites modulate several pathogenic mechanisms underlying metabolic syndrome, including oxidative stress, chronic inflammation, insulin resistance, endothelial dysfunction, and dysregulated lipid metabolism [30,31].

In plants, they function as important secondary metabolites that provide a defense against herbivores, insects, and microbial pathogens, thereby contributing to plant survival and adaptation. Beyond their ecological significance, ellagitannins have attracted considerable scientific interest because of their broad spectrum of biological activities, including antioxidant, anti-inflammatory, antimicrobial, antifungal, anticancer, cardioprotective, and antidiabetic effects [29,32].

Increasing evidence from experimental and clinical studies suggests that ellagitannins modulate multiple interconnected pathways involved in the pathogenesis of MetS, including oxidative stress, chronic low-grade inflammation, insulin resistance, endothelial dysfunction, and dysregulated lipid metabolism [29,30,31,32,33]. Their ability to simultaneously influence several molecular targets has positioned them as promising multitarget therapeutic agents for the prevention and management of MetS and its associated disorders. Accordingly, this section reviews the pharmacological activities and therapeutic prospects of pure ellagitannin compounds in the management of metabolic syndrome-related disorders. It further critically and comparatively analyzes the pharmacological profile of the reported ellagitannins, providing insights into the efficacy and possible synergistic application of natural ellagitannins in managing metabolic syndrome-related disorders. This is the first time that 17 pure ellagitannin compounds have been comprehensively discussed in a review. The review shows the broad pharmacological spectrum of different ellagitannins in the context of metabolic syndrome. It, therefore, provides a pre-clinical insight into their therapeutic prospects, whether individually, synergistically or as adjunctive therapies.

**Figure 1:**
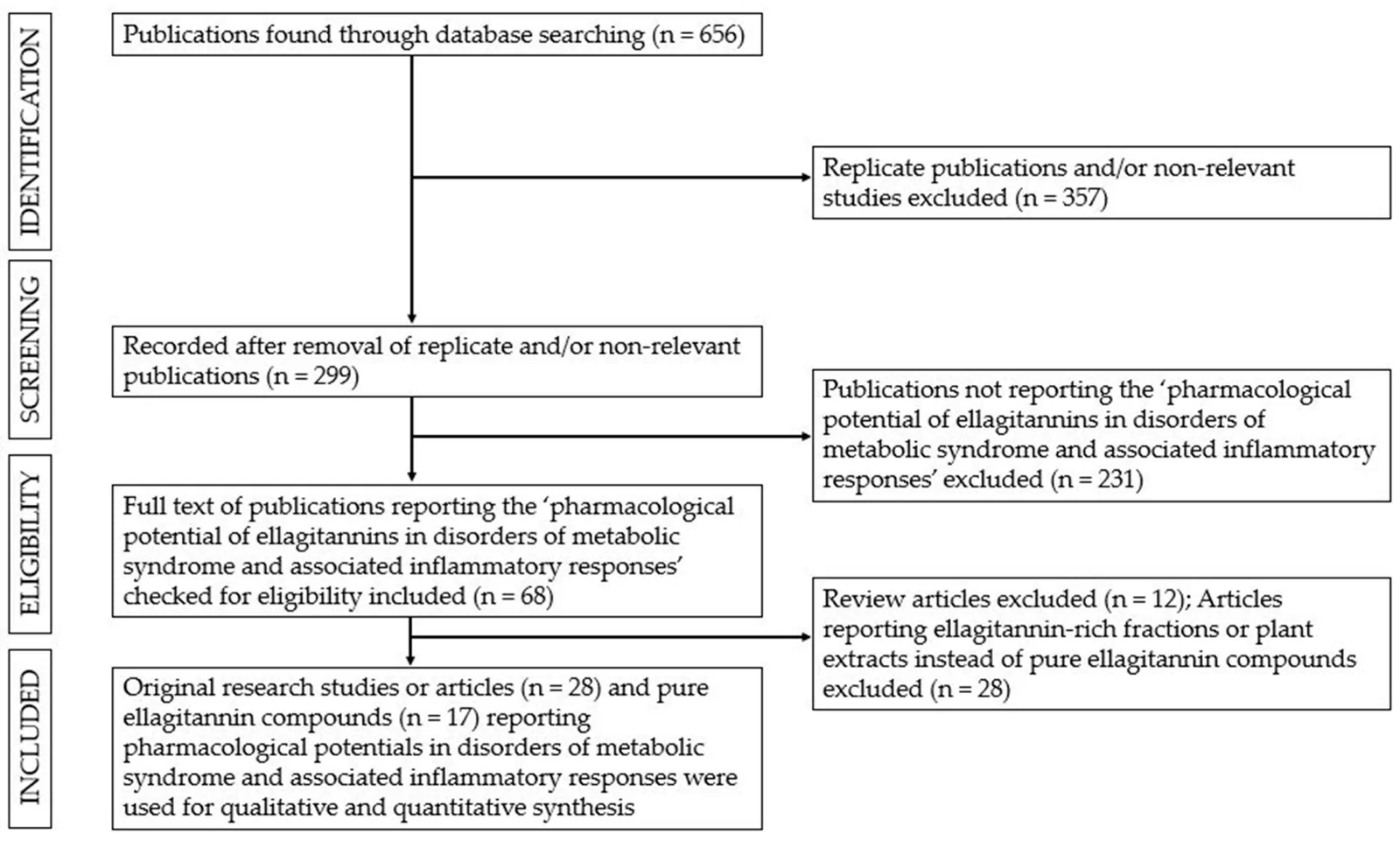
Flow diagram showing the study selection process.

A comprehensive literature search was conducted using several scientific databases and search engines, including, but not limited to, PubMed, Google Scholar, Scopus, and ScienceDirect. The search aimed to identify the peer-reviewed published literature on the “pharmacological potential of ellagitannins in disorders of metabolic syndrome and associated inflammatory responses” that was published in English, as shown in the study selection process (Figure 1). The search was done from February 2026 to June 2026. The search terms used included the following: ‘Bioactivity of ellagitannins’; ‘ellagitannins and metabolic syndrome’, antidiabetic ellagitannins’, ‘ellagitannins and type 2 diabetes’, ‘ellagitannins and glucose metabolism’, ‘ellagitannins and lipid metabolism’, ‘ellagitannins and insulin resistance’, ‘ellagitannins and insulin sensitivity’, ‘ellagitannins and insulin secretion’, ‘ellagitannins and insulin signalling’, ‘ellagitannins and blood glucose’, ‘ellagitannins and glucose uptake’, ‘ellagitannins and carbohydrate digesting enzymes’, ‘ellagitannins and blood pressure’, ‘ellagitannins and hypertension’, ‘ellagitannins and renin inhibition’, ‘ellagitannins and angiotensin-converting enzyme inhibition’, ‘Ellagitannins and enzyme inhibition’, ‘ellagitannins and spontaneously hypertensive rats’, ‘ellagitannin and dyslipidaemia’, ‘ellagitannin and blood lipids’, ‘ellagitannin and obesity’, ‘ellagitannins and cardiovascular disease’, ‘ellagitannins and oxidative stress’, ‘antioxidant ellagitannins’, ‘ellagitannins and reactive oxygen species’, ‘radical scavenging ellagitannins’, and ‘ellagitannins and inflammation’. From the results of the search, only original research studies or articles reporting the “pharmacological potential of ellagitannins in disorders of metabolic syndrome and associated inflammatory responses” were analyzed and used in synthesizing the pharmacological aspect of the review. The pharmacological information of selected studies/articles was sub-categorized according to the different disorder clusters that comprise metabolic syndrome. The pharmacological sub-categorization includes the following: antidiabetic potential; antihypertensive potential; anti-dyslipidemic, anti-obesogenic, and cardioprotective potential; antioxidant, anti-inflammatory, and tissue-protective potential; and immunomodulatory potential.

### 5.2. Structural Characteristics of Ellagitannins

**Figure 2:**
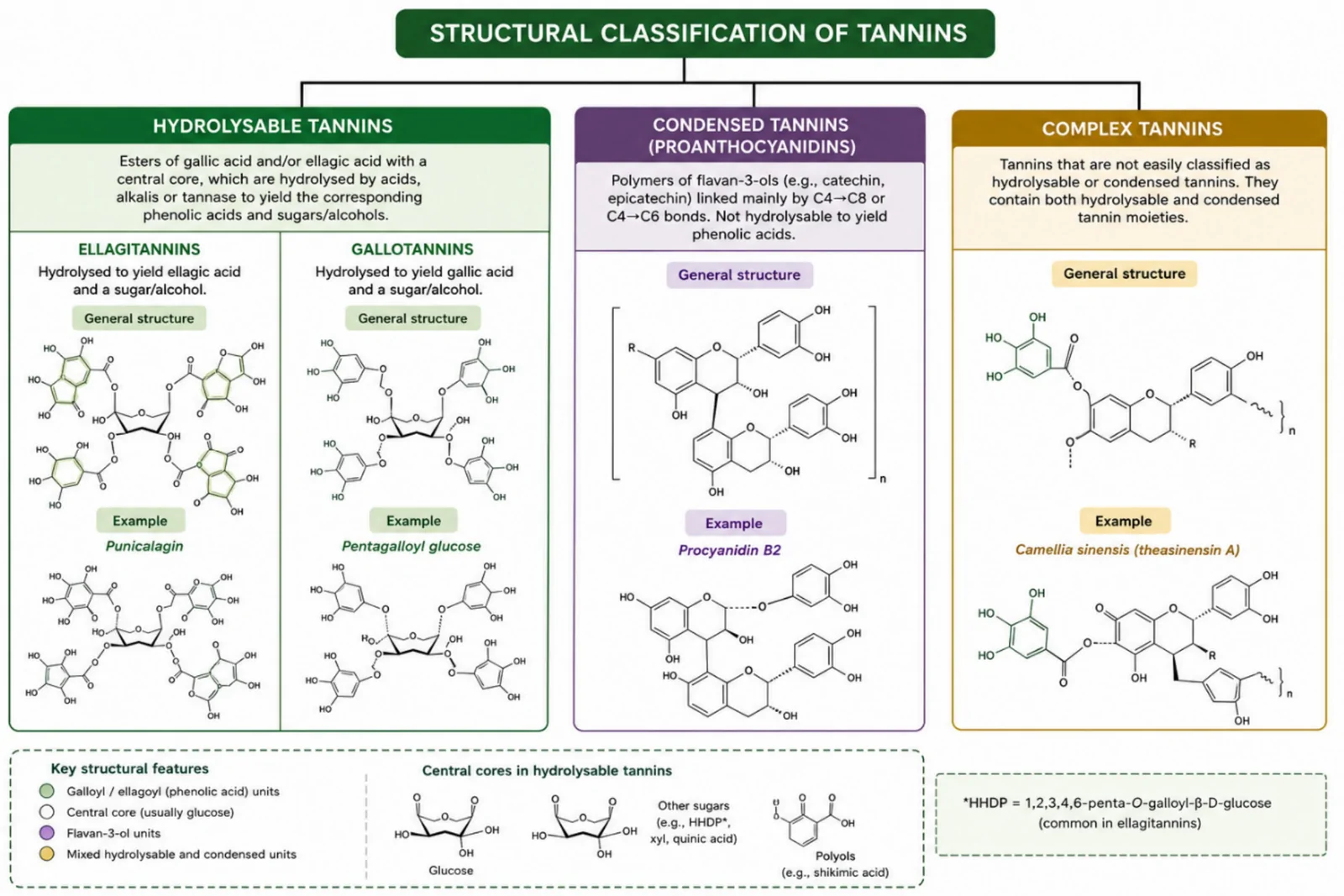
Structural classification of tannins showing the major classes of hydrolysable tannins (ellagitannins and gallotannins), condensed tannins, and complex tannins.

Tannins are a diverse class of naturally occurring, water-soluble polyphenolic compounds that are widely distributed throughout the plant kingdom. They consist of oligomeric or polymeric flavanol and phenolic acid units, which confer distinct physicochemical, sensory, and biological properties compared with their monomeric counterparts. As secondary plant metabolites, tannins play important ecological roles in plant defense against herbivores, insects, and microbial pathogens. Based on their chemical structure, tannins are broadly classified into hydrolysable tannins (comprising gallotannins and ellagitannins), condensed tannins (proanthocyanidins), complex tannins (Figure 2), which contain both hydrolysable and condensed tannin moieties, and the less common phlorotannins that are predominantly found in marine algae [29,34].

Hydrolysable tannins are characterized by a central polyol core, most commonly β-D-glucose, esterified with phenolic acids such as gallic acid or hexahydroxydiphenoyl (HHDP) groups. Based on their structural composition, hydrolysable tannins are further classified into gallotannins and ellagitannins. Gallotannins consist of one or more galloyl groups esterified to a glucose core and readily undergo hydrolysis to release gallic acid [35]. In contrast, ellagitannins are formed through the oxidative coupling of adjacent galloyl groups, generating one or more HHDP moieties that spontaneously lactonize upon hydrolysis to yield ellagic acid. The presence of HHDP groups confers greater structural complexity and chemical stability, distinguishing ellagitannins from gallotannins and contributing to their diverse biological activities [29].

Structurally, ellagitannins are esters of one or more HHDP groups linked to a monosaccharide, typically β-D-glucose. Depending on their molecular architecture, they are classified as monomeric (e.g., Geraniin, Tellimagrandin II, and Nupharin A), oligomeric (e.g., Hirtellin A, Nupharin C, and Nupharin E), or C-glycosidic ellagitannins (e.g., Vescalagin and Castalagin). Many ellagitannins undergo oxidative coupling through carbon–oxygen (C–O) or carbon–carbon (C–C) linkages, resulting in the formation of high-molecular-weight dimers and oligomers that substantially increase their structural diversity [30].

The remarkable structural diversity of ellagitannins underpins their physicochemical properties, metabolic fate, and biological activities. Variations in molecular size, degree of polymerization, hydroxylation pattern, and glycosidic linkage influence their stability, susceptibility to hydrolysis, bioavailability, antioxidant capacity, and interactions with biological targets [36]. These structural features not only determine the pharmacokinetic behavior of individual ellagitannins but also contribute to differences in their pharmacological efficacy. Consequently, understanding the structural characteristics of ellagitannins is fundamental to elucidating their therapeutic potential in the prevention and management of metabolic syndrome and its associated disorders.

### 5.3. Major Naturally Occurring Ellagitannins and Sources

Numerous ellagitannins have been identified in edible fruits, nuts, medicinal plants, and tree species. Table 1 shows the reported natural sources of the ellagitannins discussed in this review.

Among the most studied ellagitannins is Punicalagin, the predominant ellagitannin present in pomegranate (*Punica granatum* L.) peel, where it may account for up to 65.75% of the total polyphenolic content [31,55]. Punicalagin can be hydrolyzed to form Punicalin, a structurally related ellagitannin, through the loss of one HHDP group [53]. Punicalin is another major pomegranate-derived ellagitannin, predominantly found in the fruit husk, although at considerably lower concentrations than Punicalagin, accounting for approximately 2% of total husk polyphenols [55]. Another important ellagitannin is Geraniin, a highly substituted monomeric ellagitannin containing galloyl, HHDP, and dehydrohexahydroxydiphenoyl groups [29]. Geraniin occurs naturally in several plant species, including *Geranium* spp., *Phyllanthus* spp., *Euphorbia* spp., and the rind of rambutan (*Nephelium lappaceum* L.) [50]. Corilagin is a hydrolysable ellagitannin chemically identified as β-1-O-galloyl-3,6-(R)-hexahydroxydiphenoyl-D-glucose. It is widely distributed in medicinal plants, including *Phyllanthus emblica*, *Phyllanthus niruri*, *Phyllanthus reticulatus*, *Dimocarpus longan*, *Geranium wilfordii*, *Lumnitzera racemosa*, and rambutan peel (*Nephelium lappaceum*) [57]. Among the ellagitannins present in *Terminalia chebula*, Chebulagic acid and Chebulinic acid are particularly noteworthy. Chebulagic acid contains one galloyl group, one HHDP group, and a characteristic chebuloyl moiety that forms a macrocyclic ring structure, conferring greater molecular rigidity and influencing its interactions with biological targets [29]. Chebulinic acid contains three galloyl groups and one chebuloyl group, resulting in a more flexible molecular conformation that facilitates diverse interactions with enzymes and cellular targets [58].

### 5.4. Absorption and Metabolism of Ellagitannins

Despite their promising biological activities, the therapeutic efficacy of ellagitannins is strongly influenced by their limited bioavailability and extensive metabolism within the gastrointestinal tract. Owing to their large molecular size, high polarity, and complex polymeric structure, intact ellagitannins are poorly absorbed across the intestinal epithelium. Consequently, their biological effects depend largely on their metabolic conversion into smaller, more readily absorbed compounds [32].

**Figure 3:**
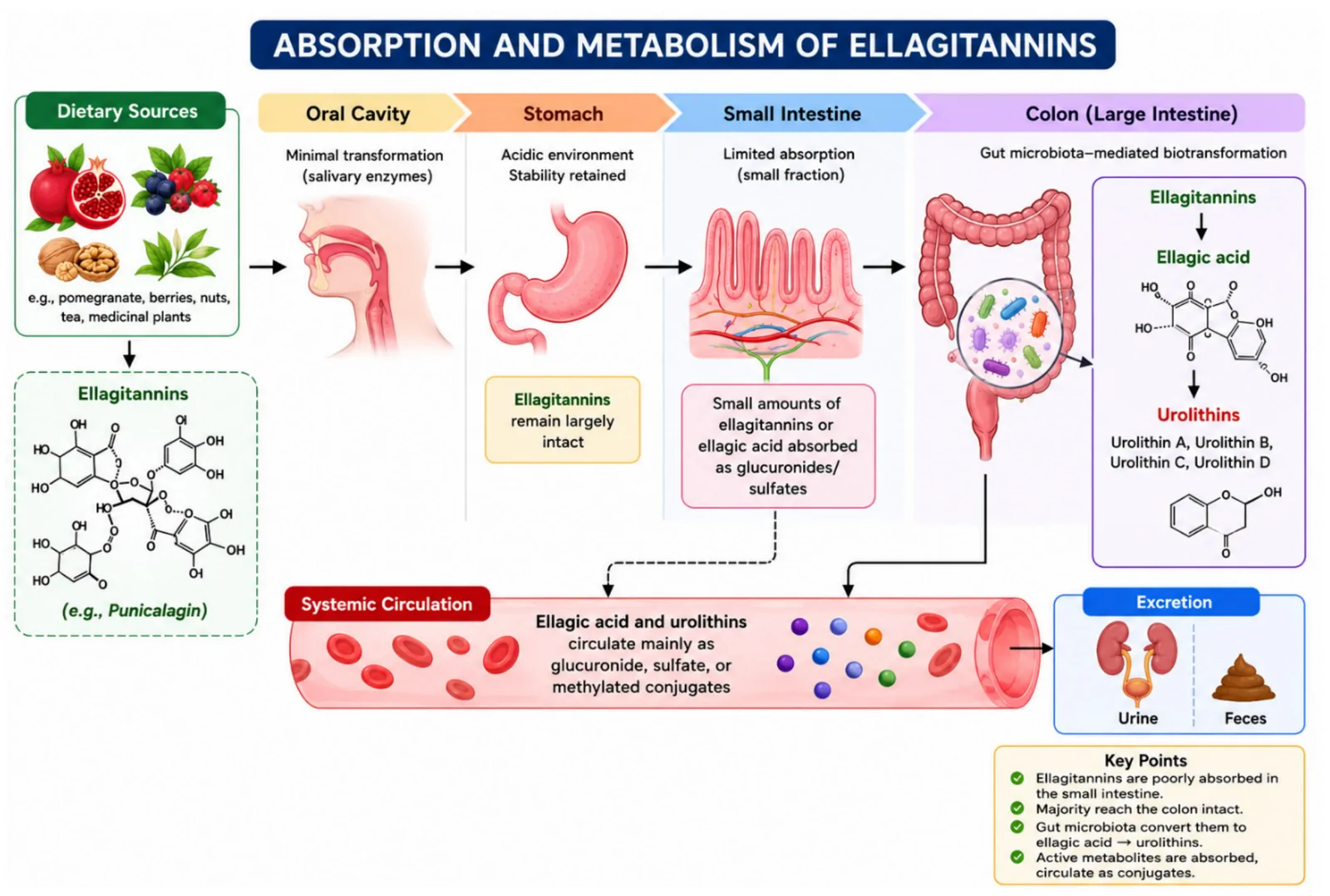
Absorption and metabolism of ellagitannins.

Following ingestion, ellagitannins undergo acid- and esterase-mediated hydrolysis within the stomach and small intestine, releasing HHDP groups from the glucose backbone (Figure 3). The liberated HHDP spontaneously undergoes intramolecular lactonization to form ellagic acid, the principal intermediate metabolite of dietary ellagitannins [29]. Although ellagic acid exhibits potent antioxidant properties, its poor aqueous solubility and limited intestinal permeability restrict its systemic bioavailability [59].

Within the colon, ellagic acid is further metabolized by specific members of the gut microbiota through sequential dehydroxylation and decarboxylation reactions, generating a series of bioactive metabolites collectively known as urolithins [45,49]. These metabolites include urolithin C, urolithin A, and urolithin B, with urolithin A generally regarded as the predominant metabolite detected in plasma and tissues following the consumption of ellagitannin-rich foods. Compared with their parent compounds, urolithins possess greater lipophilicity and are, therefore, more readily absorbed across the intestinal epithelium, enabling systemic distribution to metabolically active tissues [32].

Accumulating evidence suggests that urolithin A and urolithin B are the principal metabolites responsible for many of the health-promoting effects attributed to dietary ellagitannins [29,32].

### 5.5. Therapeutic Potential of Ellagitannins in Metabolic Disease Management

The nature of metabolic syndrome requires therapeutic agents capable of simultaneously targeting multiple pathological mechanisms. Ellagitannins have emerged as promising multitarget phytochemicals for the prevention and management of metabolic syndrome and its associated disorders. In this review, 28 original research studies or articles and 17 pure ellagitannin compounds reporting the pharmacological potential in disorders of metabolic syndrome and the associated inflammatory responses were selected and used for qualitative and quantitative synthesis, as shown in Figure 1. The in vitro and in vivo (pre-clinical) pharmacological potential of these ellagitannins in disorders of metabolic syndrome and the associated inflammatory responses is presented in Table 2 and Table 3.

#### 5.5.1. Antidiabetic Potential

Insulin resistance and impaired glucose homeostasis are central features of MetS and major contributors to the development of T2D. Increasing experimental evidence indicates that ellagitannins possess significant antidiabetic properties by simultaneously targeting multiple pathways involved in glucose metabolism, insulin signaling, and pancreatic β-cell function. Among the ellagitannins investigated, Punicalagin, Grandinin, Pedunculagin, Chebulagic acid, Geraniin, Vescalagin, Davidiin, Corilagin, Castalagin, Sanguiin H-6, Acutissimin A, and Casuarictin have demonstrated considerable therapeutic potential in both in vitro and in vivo models [31,45,53,54,60,61].

Collectively, these compounds improve glycemic control by inhibiting carbohydrate-digesting enzymes, reducing fasting blood glucose concentrations, suppressing hepatic gluconeogenesis, promoting glycogen synthesis, and enhancing insulin sensitivity through activation of the phosphatidylinositol 3-kinase/protein kinase B (PI3K/Akt) signaling pathway [31,53,54,60]. Punicalagin, Vescalagin, and Chebulagic acid have been shown to inhibit carbohydrate-digesting enzymes [31,40,56]. Maltose hydrolysis and maltase activity were downregulated by Chebulagic acid in Caco-2 cells [40], suggesting the potential of these ellagitannins to suppress postprandial glucose increase. Punicalagin exhibits an in vitro antiglycation property [31], while Geraniin reduced advanced glycation end-product (AGE) accumulation in high-fat-diet (HFD) fed rats [49], which suggests their potential to mitigate diabetic complications linked to AGE accumulation.

Furthermore, the blood glucose-lowering effects of Punicalagin, Grandinin, Chebulagic acid, Corilagin, Geraniin, and Davidiin have been documented in chemical- and diet-induced experimental animal models of diabetes and insulin resistance [40,45,51,54,57]. The blood glucose-lowering effect of Grandinin was more effective than that of Grandinin [51], a known sulfonylurea class of antidiabetic drugs. Mechanistic studies suggest that the blood glucose-lowering activities of Punicalagin, Chebulagic acid, and Geraniin are mediated through mechanisms such as the upregulation of glucose uptake and insulin signaling. Both Punicalagin and Chebulagic acid have been shown to promote glucose uptake in L6-myotubes and 3T3-L1 adipocytes, respectively [31,41], while a 4-week oral administration of Geraniin improved insulin resistance (HOMA-IR) in HFD-fed rats [48]. In HFD/STZ-induced type 2 diabetic animals, Punicalagin and Chebulagic acid exerted glycemic control effects by increasing the expression of insulin receptor (IR), insulin receptor substrate-1 (IRS-1), protein kinase B (Akt), and glucose transporter type 4 (GLUT4), thus improving insulin signaling [54,63]. These proteins play a significant role in insulin-mediated glucose uptake in peripheral tissues [64]. Thus, their upregulation by Punicalagin and Chebulagic acid strongly contributes to the glucose uptake modulatory effect of the ellagitannins [31,41]. Furthermore, the observed upregulatory potential of Punicalagin on insulin signaling proteins (Akt and PI3K) in HFD/STZ-induced mice was accompanied by modulatory action on gluconeogenic and glycogenic enzymes [54]. At an oral dose of 200 mg/kg, Punicalagin reduced hepatic expression of phosphoenolpyruvate carboxykinase, an enzyme that catalyzes a rate-limiting step of the gluconeogenic pathway [65]. Concomitantly, it reduced hepatic activation of glycogen synthase kinase-3, resulting in an increased expression of hepatic glycogen synthase and elevated glycogen content [54]. A similar effect was also observed in Punicalagin-treated HepG2 cells that were exposed to a high glucose concentration (30 mM) for 48 h, indicating that Punicalagin exerts glycemic control via suppressing gluconeogenesis and stimulating glycogenesis. Notably, Punicalagin was more effective than metformin in an animal model, suggesting that it may be a promising nutraceutical for clinical investigation in type 2 diabetic patients.

Beyond improving insulin sensitivity, ellagitannins have been shown to have modulatory potential on insulin secretion. A previous study has shown that Pedunculagin, Vescalagin, Castalagin, Sanguiin H-6, Acutissimin A, and Casuarictin dose-dependently induced glucagon-like peptide-1 (GLP1) secretion in STC1 cells [37]. GLP1 is a known incretin hormone that enhances glucose-dependent insulin secretion from pancreatic β-cells [66], suggesting that these ellagitannins have the potential to exert insulin stimulatory effect, thus potentiating glycemic control.

Overall, the available evidence suggests that ellagitannins exert antidiabetic effects through complementary mechanisms involving enhancement of insulin signaling and secretion, improvement of glucose transport and glycemic control, mitigation of diabetic complications, and inhibition of carbohydrate digestion. Their ability to simultaneously modulate multiple pathways highlights their potential as promising multitarget therapeutic agents for the management of type 2 diabetes-related metabolic disorder.

#### 5.5.2. Antihypertensive Potential

Hypertension is a major component of MetS and a significant risk factor for cardiovascular disease, stroke, and chronic kidney disease. The development of hypertension in MetS is closely associated with insulin resistance, endothelial dysfunction, oxidative stress, chronic inflammation, and overactivation of the renin–angiotensin–aldosterone system [11]. Increasing evidence indicates that several ellagitannins, including Geraniin, Corilagin, Punicalagin, Castilagin and Chebulinic acid, exhibit antihypertensive properties by targeting multiple mechanisms involved in blood pressure regulation [47,48,49]. A study has shown that Corilagin, Punicalagin, Castilagin and Chebulinic acid dose-dependently reduced arterial blood pressure in spontaneously hypertensive rats (SHRs) [39].

A principal mechanism underlying the antihypertensive activity of these compounds is the inhibition of ACE, which suppresses the conversion of angiotensin I to the potent vasoconstrictor angiotensin II. Consequently, ACE inhibition reduces peripheral vascular resistance, promotes vasodilation, and lowers systemic blood pressure [23]. Experimental studies have demonstrated that Geraniin and Corilagin possess potent ACE inhibitory activity, which could influence their reported blood pressure-lowering ability in SHRs and HFD-fed rats [39,44,47,49]. Importantly, the documented antihypertensive potential of ellagitannins should take into consideration differences in administration route, dose range, experimental model and duration of treatment.

Among the compounds evaluated by intravenous administration in SHRs, Chebulinic acid exhibited the greatest apparent acute potency, with an ED_50_ of 3.2 ± 0.9 mg/kg. A single intravenous administration of Chebulinic acid at 0.5–10 mg/kg produced a dose-dependent reduction in arterial blood pressure [39]. Punicalagin also produced a dose-dependent antihypertensive response over 0.5–15 mg/kg, but with a higher ED_50_ of 5.4 ± 1.1 mg/kg, suggesting lower acute potency than Chebulinic acid under the same experimental conditions [39]. Castalagin and Corilagin displayed comparable, although somewhat weaker, acute effects, with ED_50_ values of 6.3 ± 1.2 and 6.7 ± 1.4 mg/kg, respectively. Thus, based on ED_50_ values, the approximate order of acute blood-pressure-lowering potency was Chebulinic acid > Punicalagin > Castalagin ≈ Corilagin. These findings are particularly noteworthy because the compounds were evaluated under comparable conditions, making the ED_50_ values useful for assessing their relative acute antihypertensive activity.

The antihypertensive activity of Geraniin appears particularly interesting because, unlike the compounds for which the available evidence is primarily based on intravenous administration, geraniin has demonstrated activity following oral administration. A single oral dose of 5 mg/kg body weight significantly reduced both systolic and diastolic blood pressure in SHRs [47]. This finding suggests that Geraniin can exert antihypertensive effects following gastrointestinal administration, although its absorption, metabolism and contribution of circulating metabolites require further investigation. The oral activity is particularly relevant from a translational perspective because compounds intended for nutritional or pharmacological applications would generally be administered orally. In fact, intestinal metabolic products of ellagitannins, urolithins A and B, had stronger ACE inhibitory activity (75%) compared to Geraniin (55%) at 0.0001 mM [49], which further explains the potent antihypertensive property of orally administered Geraniin. Possibly, orally administered Geraniin is metabolized into urolithins, which potentiate a stronger ACE inhibitory action and blood pressure-lowering effect.

Furthermore, Geraniin inhibited ACE with an IC_50_ of 0.4 mM in the study of Ueno et al. (1988) [44], while Lin et al. (2008) [47] reported an IC_50_ of 13.22 µM. Looi et al. (2021) [49] subsequently reported approximately 55% ACE inhibition at 0.0001 mM. Although these values collectively support ACE-inhibitory potential, the substantial differences in reported potency should be interpreted cautiously because in vitro ACE inhibition is highly dependent on assay conditions, substrate concentration, enzyme source and experimental methodology. Therefore, the reported IC_50_ values should not be directly ranked across studies without methodological normalization. Nevertheless, inhibition of ACE provides a plausible mechanistic explanation for the blood-pressure-lowering effect of Geraniin.

Further evidence suggests that geraniin may exert antihypertensive effects through mechanisms extending beyond ACE inhibition. In high-fat-diet-fed Sprague–Dawley rats, four weeks of oral Geraniin administration (3.125–100 mg/kg) reduced the mean systolic blood pressure, with notable effects reported at 3.125 mg/kg. This was accompanied by reductions in serum insulin and renin concentrations and decreased accumulation of advanced glycation end products (AGEs) [48]. The reduction in renin is particularly relevant because renin initiates the RAAS cascade by converting angiotensinogen to angiotensin I [11]. Suppression of renin could, therefore, complement ACE inhibition and provide a more comprehensive attenuation of RAAS-mediated hypertension. Furthermore, the reduction in AGEs may be relevant to vascular protection because excessive AGE accumulation can promote oxidative stress, endothelial dysfunction and vascular stiffness, all of which can contribute to hypertension. The concomitant reduction in serum insulin also raises the possibility that geraniin may indirectly improve blood pressure through metabolic effects, particularly in the context of diet-induced metabolic dysfunction.

Although the available evidence is promising, most studies have been conducted on experimental models, with limited clinical evidence available. Also, differences in the dose, duration of treatment, experimental models and bioavailability of ellagitannins should be considered when comparing the relative potency of individual ellagitannins. Well-designed human clinical trials are required to establish the long-term efficacy, optimal dosage, and safety of ellagitannins as antihypertensive agents in individuals with metabolic syndrome.

#### 5.5.3. Anti-Dyslipidemic, Anti-Obesogenic, and Cardioprotective Potential

Dyslipidemia and obesity are hallmark features of MetS that contribute substantially to CVD by promoting insulin resistance, endothelial dysfunction, chronic inflammation, and atherosclerosis. Increasing evidence suggests that several ellagitannins, particularly Geraniin, Corilagin, Punicalagin, and Oenothein B, exhibit lipid-lowering properties through the modulation of lipid metabolism and energy homeostasis, with evidence supporting anti-dyslipidemic, anti-obesogenic and cardioprotective properties [50,52,54,57].

In HFD/STZ-induced type 2 diabetic C57BL/6J mice, four weeks of oral administration at 100–200 mg/kg of Punicalagin dose-dependently reduced serum triglyceride (TG) and total cholesterol (TC) concentrations [54]. The lipid-lowering effect occurred alongside the suppression of hepatic phosphoenolpyruvate carboxykinase (PEPCK), increased glycogen synthesis through activation of glycogen synthase (p-GS) and inhibition of glycogen synthase kinase-3β (p-GSK3β), and enhanced PI3K/p-Akt signaling. These findings indicate that the anti-dyslipidemic effect of punicalagin may be closely associated with the restoration of insulin signaling and improved hepatic glucose metabolism. Since impaired insulin signaling promotes excessive hepatic gluconeogenesis and contributes to abnormalities in lipid metabolism, activation of the PI3K/Akt pathway could indirectly reduce the metabolic drive toward dyslipidemia [67]. Furthermore, suppression of IL-6, TNF-α and MCP-1 suggests that punicalagin may attenuate inflammation-associated metabolic dysfunction. This is particularly relevant to cardioprotection because chronic low-grade inflammation, dyslipidemia and insulin resistance are interconnected drivers of endothelial dysfunction and cardiovascular injury [9].

Geraniin and Chebulagic acid demonstrated pronounced anti-obesogenic and anti-dyslipidemic effects that appear to be associated with modulation of signaling and glucose metabolism [41,50]. Chebulagic acid enhanced pre-adipocyte differentiation by activating PPARγ signaling and increasing adiponectin levels, GLUT-4 and C/EBP-α expression, and glucose uptake in the differentiated adipocytes [41]. The activation of PPARγ, accompanied by increased C/EBP-α, suggests a coordinated transcriptional regulation of adipogenesis and lipid storage by Chebulagic acid [68]. Furthermore, the enhanced adiponectin production may improve insulin sensitivity and facilitate metabolic homeostasis [69], which may be implicated in the observed enhanced GLUT-4 signaling and cellular glucose uptake [68]. Collectively, these effects indicate that chebulagic acid may enhance insulin-mediated metabolic signaling through coordinated PPARγ/C/EBP-α–adiponectin and GLUT4 pathways, thereby improving glucose utilization and lipid metabolism. In HFD-fed Sprague–Dawley rats, relatively low doses of Geraniin (5 and 10 mg/kg) administered for four weeks reduced plasma TG, LDL-cholesterol and TC while increasing HDL-cholesterol [50]. These effects were accompanied by reduced fasting glucose, improved HOMA-IR and glucose tolerance, and increased circulating insulin and adiponectin. The elevation of adiponectin is a notable effect because adiponectin has been reported to enhance fatty acid oxidation, improve insulin sensitivity and exert anti-inflammatory and cardiometabolic effects [70]. The study of Moorthy et al. (2022) [50] further demonstrated that Geraniin also increased PPARα expression, a nuclear receptor that promotes hepatic and systemic fatty acid oxidation and facilitates lipid clearance. Thus, the lipid-lowering activity of geraniin may involve a simultaneous improvement of insulin sensitivity and stimulation of fatty acid catabolism.

Corilagin also displayed appreciable anti-dyslipidemic and anti-obesogenic potential. Fifteen weeks of oral administration to HFD-fed C57BL/6 mice dose-dependently decreased TG, TC, LDL-cholesterol, free fatty acids and fatty acid-binding protein 4 (FABP4), while increasing HDL-cholesterol [57]. These changes were accompanied by reductions in fasting glucose and insulin, improved glucose and insulin tolerance, enhanced insulin sensitivity and decreased HOMA-IR. Particularly noteworthy was the reduction of FABP4, an adipocyte-derived lipid-binding protein associated with increased fatty acid flux, insulin resistance and cardiometabolic risk [71]. The reduction in circulating free fatty acids may, therefore, indicate improved regulation of adipose lipid mobilization and reduced ectopic lipid deposition. In the study of Liao et al. (2022) [57], Corilagin also reduced ALT and AST levels and decreased the non-alcoholic fatty liver disease (NAFLD) ‘Activity Score’, demonstrating protection against hepatic lipid-associated injury. These effects suggest that Corilagin may interrupt adipose tissue–liver axis pathologies, whereby excessive adipose-derived fatty acids contribute to hepatic steatosis, insulin resistance and systemic dyslipidemia.

The effects of Oenothein B appear to be more anti-obesogenic, although the available evidence is in a non-mammalian model. Treatment with 640 µM Oenothein B reduced fat accumulation in glucose-exposed C. elegans, an effect associated with downregulation of the genes involved in lipogenesis and fatty acid desaturation [52]. This observation suggests that Oenothein B may act directly on the pathways controlling de novo lipid synthesis and fatty acid composition, thereby limiting lipid storage. Although this finding provides mechanistic support for an anti-obesogenic effect, its translational significance should be interpreted cautiously because the study was performed in C. elegans and at a relatively high concentration.

The available evidence indicates that ellagitannins may target the metabolic syndrome at multiple interconnected levels, including lipid synthesis and oxidation, adipose tissue function, insulin signaling, hepatic lipid accumulation and inflammation. By improving lipid metabolism, reducing adipose tissue accumulation, and alleviating metabolic dysfunction, ellagitannins may contribute to the prevention of atherosclerosis and other cardiovascular complications associated with MetS [33]. However, most evidence remains preclinical. Further pharmacological and mechanistic investigations and clinical studies are required to establish their direct cardioprotective effects and determine their therapeutic value in cardiovascular diseases.

#### 5.5.4. Antioxidant, Anti-Inflammatory, and Tissue-Protective Potential

Oxidative stress and chronic low-grade inflammation are fundamental drivers of MetS, contributing to insulin resistance, endothelial dysfunction, dyslipidemia, hypertension, and progressive tissue injury. Consequently, therapeutic agents capable of simultaneously attenuating oxidative damage and inflammatory responses have attracted considerable attention. Among the pharmacological activities reported for ellagitannins, antioxidant, anti-inflammatory, and tissue-protective effects represent the most extensively investigated. Experimental studies have demonstrated that several ellagitannins, including Geraniin, Punicalagin, Punicalin, Vescalagin, Chebulagic acid, Chebulinic acid, Corilagin, and Amariin, exert potent protective effects through multiple complementary mechanisms [28,31,38,47,53,55,56,61].

The antioxidant activity of ellagitannins is attributed to their polyphenolic structure, which enables them to directly neutralize reactive oxygen species (ROS) and other free radicals. Punicalagin, Punicalin, Vescalagin, Grandinin, Chebulinic acid and Geraniin have demonstrated strong radical-scavenging activity against DPPH, ABTS, hydroxyl, superoxide, and oxygen radicals [28,31,43,47,51,53,55,56,61]. While Punicalagin and Punicalin exhibit greater antioxidant capacity than the reference antioxidant Trolox in several experimental assays [31,53,55,61], the computed radical scavenging IC_50_ values suggest that Chebulinic acid (IC_50_ = 12.5 µM) is the most effective radical scavenger, at least for DPPH [62]. This is, however, subject to confirmatory studies under similar experimental conditions. In addition to directly scavenging free radicals, Chebulinic acid, Chebulagic acid, Geraniin and Corilagin inhibited ferroptosis by simultaneously reducing ROS accumulation and chelating transition metals such as iron (Fe^2+^) and copper (Cu^2+^), thereby limiting metal-catalyzed oxidative damage [43,62]. Furthermore, Geraniin, Grandinin, Chebulagic acid, Chebulinic acid, Punicalin, Oenothein B, Amariin, and Punicalagin enhanced endogenous antioxidant defense systems by increasing the activities of superoxide dismutase (SOD), catalase (CAT), glutathione peroxidase (GPx), and glutathione reductase (GR), while significantly reducing lipid peroxidation [28,31,38,43,51,52,53,62,63]. Importantly, the in vivo systemic or tissue antioxidant activities shown by Punicalagin, Punicalin, Grandinin, and Geraniin were observed in various experimental disease models or pathologies such as lipid-induced oxidative stress [53], STZ-induced diabetes [51], acetaminophen- and carbon tetrachloride-induced hepatotoxicity [43], HFD-induced metabolic disorder and oxidative stress [28], and HFD/STZ-induced T2D, suggesting that these ellagitannins could mitigate oxidative stress-mediated complications and tissue damage in metabolic diseases.

Beyond their antioxidant properties, Punicalagin, Punicalin, Chebulinic acid, Chebulagic acid, and Geraniin exhibit potent anti-inflammatory activities through the suppression of multiple pro-inflammatory signaling pathways [42,53,54,60]. Punicalagin and Punicalin reduce the production of inflammatory cytokines, including interleukin-6 (IL-6), tumor necrosis factor-α (TNF-α), and monocyte chemoattractant protein-1 (MCP-1), in both renal and intestinal tissues [53,54,60]. Similarly, Chebulagic acid suppresses key inflammatory mediators such as IL-1β, IL-6, TNF-α, and nuclear factor-kappa B (NF-κB) [63], whereas Chebulinic acid and Geraniin inhibit nitric oxide (NO) production and downregulate the expression of inducible nitric oxide synthase (iNOS) and cyclooxygenase-2 (COX-2), thereby limiting inflammatory tissue damage [42]. In obese animal models, geraniin has also been shown to suppress systemic endotoxemia, further highlighting its ability to attenuate the chronic inflammation associated with metabolic dysfunction [50].

The combined antioxidant and anti-inflammatory properties of ellagitannins translate into substantial tissue-protective effects across multiple organs affected by MetS. Ex vivo, Geraniin and Amariin protected liver tissue slices against ethanol-induced cytotoxicity through reduced apoptosis (Bax:Bcl-2 ratio), lactate dehydrogenase enzyme release, lipid peroxidation, protein oxidation and oxidative damage of DNA but increased antioxidant enzymes (CAT, SOD, GPx, and GR) activity [38]. Hepatoprotective studies have demonstrated that Punicalagin, Corilagin, and Chebulinic acid protect against diet- and chemically induced liver injury by reducing hepatocellular apoptosis, preserving liver architecture, decreasing liver enzymes, alanine aminotransferase (ALT) and aspartate aminotransferase (AST) activities, and improving NAFLD indexes [43,54,57].

The hepatoprotective potential of Chebulinic acid is particularly noteworthy, as its concomitant activities across multiple experimental models indicate a multifaceted pharmacological profile encompassing antioxidant, anti-inflammatory, and hepatoprotective effects [43]. In t-BHP-challenged L-02 hepatocytes, Chebulinic acid dose-dependently suppressed intracellular ROS accumulation and LDH leakage, while enhancing ERK, JNK and p38 MAPK phosphorylation and activating the Nrf2/HO-1 antioxidant pathway [43], suggesting coordinated regulation of stress-response and cytoprotective signaling [72]. This antioxidant capacity was corroborated in vivo, where oral administration attenuated CCl_4_-induced hepatotoxicity by reducing lipid peroxidation and ALT/AST elevations, restoring SOD activity, improving hepatic histopathology, and activating Nrf2/HO-1 signaling [43]. Its ability to ameliorate acetaminophen-induced hepatotoxicity in transgenic zebrafish further supports its broad hepatoprotective action [43]. Collectively, these findings suggest that chebulinic acid protects hepatic tissue through ROS suppression, reinforcement of endogenous antioxidant defenses, modulation of MAPK signaling, and preservation of cellular membrane integrity, thereby limiting oxidative injury and subsequent tissue damage.

Reno-protective effects have also been reported, with Punicalagin preventing diabetes-induced renal injury and Grandinin and Chebulagic acid reducing blood urea nitrogen and serum creatinine concentrations, indicating preservation of renal function [51,54,63]. In addition, Punicalagin and Punicalin improve intestinal barrier integrity and attenuate histopathological damage induced by oxidative dietary insults [53]. Beyond metabolic tissues, Geraniin and Furosin have demonstrated significant analgesic and antinociceptive activities, exhibiting greater efficacy than aspirin and acetaminophen in experimental pain models [46].

Collectively, these findings demonstrate that ellagitannins exert broad cytoprotective effects by simultaneously targeting oxidative stress, inflammation, ferroptosis, and tissue injury. Their ability to preserve the structural and functional integrity of metabolically active organs, including the liver, kidneys, pancreas, and intestine, further supports their potential as multitarget therapeutic agents for preventing the progression and complications of metabolic syndrome.

#### 5.5.5. Immunomodulatory Potential

Immune dysregulation is increasingly recognized as a key contributor to the chronic low-grade inflammation that underlies metabolic syndrome (MetS). Persistent activation of immune cells promotes the production of pro-inflammatory cytokines, which exacerbate insulin resistance, endothelial dysfunction, and tissue injury. Consequently, therapeutic agents capable of restoring immune homeostasis may provide additional benefits in the management of MetS.

Although comparatively fewer studies have investigated the immunomodulatory effects of ellagitannins, the available evidence suggests that these compounds can modulate immune responses by suppressing excessive inflammation while promoting anti-inflammatory signaling. Among the ellagitannins investigated, Punicalagin, Punicalin, and Chebulagic acid have demonstrated promising immunomodulatory activities in both in vitro and in vivo studies [53,63].

In human peripheral blood mononuclear cells (PBMCs), Punicalagin and Punicalin exert dose-dependent immunomodulatory effects by reducing the production of pro-inflammatory cytokines, including tumor necrosis factor-α (TNF-α), IL-6, and IL-8, while simultaneously increasing the secretion of the anti-inflammatory cytokine, IL-10 [53]. Similarly, Chebulagic acid has been shown to promote systemic immune homeostasis in vivo through suppression of pro-inflammatory cytokine expression accompanied by increased IL-10 production, thereby contributing to the resolution of chronic inflammation [63].

Collectively, these findings suggest that ellagitannins exert immunomodulatory effects by shifting the immune response from a pro-inflammatory to a more regulated anti-inflammatory state. Given the central role of chronic inflammation in the pathogenesis of MetS, modulation of immune function may represent an additional mechanism through which ellagitannins improve metabolic homeostasis and reduce disease progression [73]. However, the current evidence remains limited, and further mechanistic studies and clinical investigations are required to fully elucidate their immunomodulatory potential in metabolic syndrome.

Overall, the available evidence demonstrates that ellagitannins exert multifaceted therapeutic effects across multiple components of metabolic syndrome. Rather than targeting a single metabolic abnormality, these compounds simultaneously modulate glucose metabolism, lipid homeostasis, blood pressure regulation, oxidative stress, chronic inflammation, immune responses, and tissue integrity. This multitarget mode of action distinguishes ellagitannins from many conventional pharmacological therapies, which typically address the individual components of the syndrome, thereby highlighting their potential as promising supplementary agents for the prevention and management of metabolic syndrome. Moving forward, transitioning these robust in vitro and in vivo findings into rigorous clinical trials will be essential to fully validate their therapeutic efficacy in human populations.

### 5.6. Comparative Assessment of the Pharmacological Potential of Ellagitannins in Metabolic Syndrome-Related Diseases

**Figure 4:**
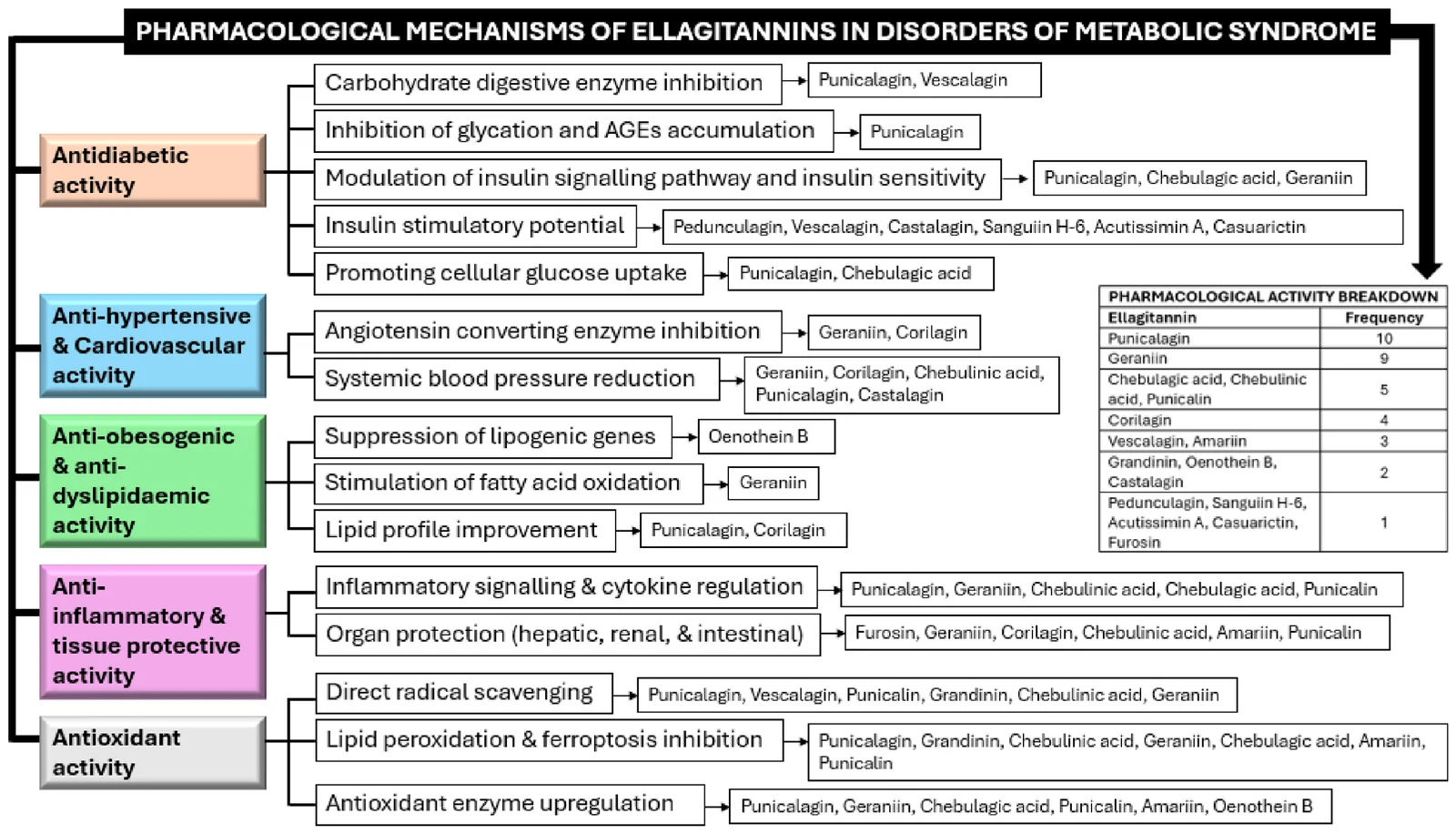
Summarized pharmacological mechanism of ellagitannins in disorders of metabolic syndrome.

The available evidence demonstrates considerable variation in the pharmacological profiles and strength of evidence among the ellagitannins investigated. Although all compounds exhibit beneficial biological activities relevant to MetS, the breadth of therapeutic effects and the extent of experimental validation differ substantially. Figure 4 shows a summarized pharmacological mechanism of ellagitannins in disorders of metabolic syndrome, highlighting the variation in their pharmacological profiles.

Among the ellagitannins reviewed, punicalagin demonstrates the broadest spectrum of pharmacological activity. Evidence from both in vitro cellular models and in vivo animal studies consistently supports its antidiabetic, antioxidant, anti-inflammatory, tissue-protective, and immunomodulatory properties. Mechanistically, Punicalagin improves insulin signaling through upregulation of IR, IRS-1, Akt, and GLUT4, suppresses oxidative stress and inflammatory cytokine production, protects hepatic, renal, and intestinal tissues, and improves dyslipidemia, making it one of the most comprehensively investigated ellagitannins for MetS [31,53,54,60,61].

Similarly, Geraniin exhibits extensive multitarget activity. Beyond improving glucose homeostasis, Geraniin attenuates diet-induced obesity, stimulates hepatic fatty acid oxidation, improves dyslipidemia, lowers systemic blood pressure, suppresses endotoxemia, inhibits AGE accumulation, and possesses potent antioxidant and anti-inflammatory activities. These complementary actions suggest that geraniin targets several interconnected pathological mechanisms underlying MetS rather than a single metabolic abnormality [28,38,44,46,47,48,49,50].

Chebulagic acid also demonstrates considerable therapeutic potential by simultaneously improving insulin sensitivity, enhancing glucose uptake through increased expression of GLUT4, IR, IRS-1, and Akt, reducing postprandial glucose absorption, suppressing oxidative stress, inhibiting ferroptosis, and protecting renal function [40,41,62,63]. Likewise, Corilagin exhibits broad biological activity through improvements in lipid metabolism, blood pressure regulation, antioxidant defense, anti-inflammatory responses, and hepatoprotection [39,44,57].

A distinct group comprising Pedunculagin, Vescalagin, Castalagin, Sanguiin H-6, Acutissimin A, and Casuarictin shares a common mechanism involving stimulation of GLP-1 secretion in STC1 cells, suggesting their potential to enhance glucose-dependent insulin secretion. However, compared with Punicalagin and Geraniin, these compounds have been investigated in fewer experimental models, and their broader therapeutic potential remains to be established [37].

Overall, Punicalagin and Geraniin currently possess the strongest preclinical evidence, supported by multiple independent studies employing both cellular and animal models that consistently demonstrate beneficial effects across several components of MetS. Also, there is more in-depth mechanistic pre-clinical or in vivo evidence of the pharmacological potential of Punicalagin and Geraniin in MetS (Table 3). Chebulagic acid, Chebulinic, Corilagin, and Punicalin also show considerable promise due to appreciable mechanistic pre-clinical pharmacological evidence but require further validation in diverse experimental systems and more comprehensive molecular and mechanistic investigations.

Although the collective evidence highlights ellagitannins as promising multitarget therapeutic agents, most findings remain confined to preclinical studies, underscoring the need for well-designed human clinical trials to confirm their efficacy, establish optimal dosing strategies, and determine long-term safety. Additionally, the tentative ranking of these ellagitannins should be interpreted with caution since the experimental conditions, including the experimental models, dose, duration and route of treatment/administrations, varied across the different studies. Also, it would be worthwhile to investigate potential synergistic interactions among ellagitannins when administered in combination, which may enhance therapeutic efficacy and produce broader multifaceted pharmacological effects.

### 5.7. Safety, Toxicology and Regulatory Information

Current evidence suggests that naturally occurring ellagitannins possess a favorable safety profile and are generally well-tolerated at doses evaluated in experimental studies. In vitro and preclinical investigations have consistently demonstrated no or low acute toxicity, with no significant adverse effects on major organs, including the liver, kidneys, and pancreas, following administration of several ellagitannins such as Punicalagin, Geraniin, Corilagin, and Chebulagic acid [28,41,43,45,54,60]. For example, Chebulagic acid remained non-toxic to Caco-2 and 3T3-L1 cells even at concentrations of 500 µM, which was the same as [40] or 5-fold more than the highest tested bioactive concentration [41]. Furthermore, acute pharmacotoxicity studies in mice showed that the LD_50_ of Grandinin in mice was 316 mg/kg bw, which was 4.5-fold more than the highest bioactive or effected dose [51]. Moreover, the multitarget mechanisms of action, combined with minimal evidence of organ toxicity in experimental models, suggest that ellagitannins may represent safer therapeutic candidates than some conventional pharmacological agents used in the management of metabolic syndrome [50,54,60,63].

Despite these encouraging findings, the toxicological evaluation of ellagitannins remains incomplete. Most available evidence is derived from short-term in vitro/cell-based and animal studies, while comprehensive investigations into long-term toxicity, reproductive and developmental toxicity, genotoxicity, carcinogenicity, maximum tolerated doses, and potential herb–drug interactions are scarce. Consequently, although the current data support a favorable safety profile, additional well-designed toxicological studies are required before ellagitannins can be considered suitable for routine clinical use [45,57,74].

Purified ellagitannins have not been approved as pharmaceutical agents by major regulatory authorities, including the United States Food and Drug Administration and the European Medicines Agency, for the treatment of metabolic syndrome or its associated disorders [45,54]. Consequently, their use remains largely confined to preclinical research and early clinical investigations. In contrast, plant extracts naturally enriched in ellagitannins, particularly pomegranate-derived extracts, are widely marketed as nutraceuticals and functional food ingredients because of their antioxidant and cardiometabolic health benefits. In the United States, certain pomegranate extracts have been accepted as “Generally Recognized as Safe” for specified food applications, supporting their use as dietary ingredients rather than therapeutic drugs [31].

Overall, the available evidence indicates that ellagitannins possess an encouraging safety profile and substantial therapeutic potential. Nevertheless, challenges related to incomplete toxicological characterization, poor bioavailability, inter-individual metabolic variability, and the absence of regulatory approval remain significant barriers to their clinical translation. Addressing these limitations through rigorous toxicological assessment, pharmacokinetic optimization, and well-designed randomized clinical trials will be essential for the future development of ellagitannin-based therapeutics.

### 5.8. Limitation, Research Gap and Recommendations

Although substantial preclinical evidence demonstrates that ellagitannins possess antidiabetic, antihypertensive, antidyslipidemic, antioxidant, anti-inflammatory, immunomodulatory, and tissue-protective activities, several important limitations continue to hinder their translation into clinical practice [54,60,63]. Most available evidence is derived from in vitro experiments and animal models, while well-designed randomized controlled clinical trials evaluating purified ellagitannins in patients with metabolic syndrome remain scarce. Consequently, the long-term efficacy, optimal therapeutic dosage, pharmacokinetics, and safety of individual ellagitannins in humans have not yet been fully established [74].

Another major limitation is the poor oral bioavailability of ellagitannins. Owing to their high molecular weight and complex chemical structure, they are poorly absorbed in the gastrointestinal tract and undergo extensive metabolism by intestinal microbiota before reaching the systemic circulation [49,74]. Furthermore, considerable inter-individual variation in gut microbial composition results in different rates of urolithin production, leading to variability in therapeutic responses among individuals. Therefore, strategies that improve bioavailability and achieve more predictable pharmacokinetic profiles remain a major research priority [50,74].

Although several ellagitannins demonstrated favorable effects on dyslipidemia and hypertension, direct evidence supporting cardioprotective effects, particularly in terms of preventing myocardial injury, improving cardiac function, or reducing cardiovascular mortality, remains limited. Future studies should, therefore, investigate their effects on cardiac tissue, vascular remodeling, endothelial function, and long-term cardiovascular outcomes using clinically relevant disease models and human trials [39,48,57].

Future research should also focus on elucidating the precise molecular mechanisms through which ellagitannins regulate interconnected metabolic pathways. Multi-omics approaches, including transcriptomics, proteomics, metabolomics, and gut microbiome analyses, may provide greater insight into the complex interactions between ellagitannins, host metabolism, and microbial-derived metabolites such as urolithins [49,50].

To overcome pharmacokinetic limitations, considerable attention should be directed toward the development of bioavailability-enhancing strategies, including nanoformulations, nanoemulsions, liposomal and polymeric encapsulation systems, structural modification of ellagitannin molecules, and targeted delivery platforms [74]. Combination therapies involving ellagitannins and conventional antidiabetic or cardioprotective drugs may also provide synergistic therapeutic effects while potentially reducing drug dosage and adverse effects [54]. Standardization of ellagitannin-rich extracts, together with large-scale clinical trials evaluating efficacy, safety, pharmacokinetics, and regulatory compliance, will ultimately be essential before ellagitannins can be translated into evidence-based therapeutic interventions for metabolic syndrome [31,74].

## 6. Conclusions

Metabolic syndrome remains one of the most significant global public health challenges due to its increasing prevalence and its strong association with type 2 diabetes mellitus, cardiovascular disease, metabolic dysfunction-associated steatotic liver disease, and other chronic non-communicable diseases. Although current therapeutic strategies, including lifestyle modification, pharmacotherapy, and bariatric surgery, effectively manage individual components of the syndrome, they largely adopt a symptom-based approach and are often associated with polypharmacy, adverse effects, high healthcare costs, and poor long-term adherence. Consequently, there is a growing need for novel therapeutic agents capable of simultaneously targeting the multiple interconnected mechanisms underlying metabolic syndrome.

Ellagitannins have emerged as promising multitarget bioactive compounds owing to their diverse pharmacological properties. Experimental evidence demonstrates that these hydrolysable polyphenols exert antidiabetic, antihypertensive, antidyslipidemic, antioxidant, anti-inflammatory, immunomodulatory, and tissue-protective effects by modulating the key molecular pathways involved in insulin resistance, oxidative stress, chronic inflammation, lipid metabolism, and endothelial dysfunction. Their ability to influence multiple pathogenic mechanisms simultaneously distinguishes them from conventional therapies that primarily target individual metabolic abnormalities.

Despite these encouraging findings, the clinical translation of ellagitannins remains constrained by poor oral bioavailability, dependence on gut microbial metabolism, inter-individual variability in urolithin production, limited long-term toxicological data, and a shortage of robust human clinical trials. Future research should, therefore, focus on improving their pharmacokinetic properties through advanced drug delivery systems such as nanoformulations and encapsulation technologies, while conducting well-designed clinical studies to establish efficacy, safety, optimal dosing, and long-term therapeutic outcomes. Furthermore, potential synergistic interactions among ellagitannins when administered in combination should be investigated for enhanced therapeutic efficacy and broader multifaceted pharmacological effects.

Overall, ellagitannins represent a promising class of naturally derived multitarget therapeutic agents with the potential to complement or enhance existing strategies for the prevention and management of metabolic syndrome. Continued advances in formulation technologies, mechanistic research, and clinical evaluation will be critical for translating their considerable preclinical promise into effective evidence-based interventions for patients with metabolic syndrome.

## Figures and Tables

**Table 1:** Reported natural sources of ellagitannins discussed in this review.

Ellagitannins	Reported Natural Sources	References
Acutissimin A	*Rubus occidentalis* (seeds)	[37]
Amariin	*Phyllanthus amarus* (aerial part)	[38]
Castalagin	*Rubus occidentalis* (seeds), *Lumnitzera racemosa* (leaves)	[37,39]
Casuarictin	*Rubus occidentalis* (seeds)	[37]
Chebulagic acid	*Terminalia chebula* (fruit)	[40,41]
Chebulinic acid	*Terminalia chebula* (fruit), *Lumnitzera racemosa* (leaves)	[39,42,43]
Corilagin	*Lumnitzera racemosa* (leaves), *Phyllanthus niruri* (aerial part)	[39,44]
Davidiin	*Polygonum capitatum* (aerial part)	[45]
Furosin	*Phyllanthus sellowianus* (leaves and stems)	[46]
Geraniin	*Nephelium lappaceum* (fruit rind), *Phyllantus urinaria* (aerial part), *Phyllanthus niruri* (aerial part), *Phyllanthus amarus* (aerial part), *Phyllanthus sellowianus* (leaves and stems)	[28,38,44,46,47,48,49,50]
Grandinin	*Melaleuca quinquenervia* (leaves)	[51]
Oenothein B	*Eucalyptus globulus* (leaves)	[52]
Pedunculagin	*Rubus occidentalis* (seeds)	[37]
Punicalagin	*Punica granatum* (fruit and peel); *Lumnitzera racemosa* (leaves)	[31,39,53,54]
Punicalin	*Punica granatum* (fruit, peel and husk)	[53,55]
Sanguiin H-6	*Rubus occidentalis* (seeds)	[37]
Vescalagin	*Rubus occidentalis* (seeds), *Syzygium samarangense* (unripe fruit)	[37,56]

**Table 2:** In vitro pharmacological Potential of Ellagitannins in Disorders of Metabolic Syndrome and Associated Inflammatory Responses.

Ellagitannin	Model and Treatment	Medicinal Potential	Effect/Molecular Targets	References
Punicalagin	20, 40 and 80 mM in high-glucose-induced HepG2	Antidiabetic	• Glycogen content ↑ • glycogenic activation (p-GS and p-GSK3β) ↑ • Gluconeogenesis (PEPCK) ↓ • Insulin signaling (PI3K and p-Akt) ↑	[54]
	20, 40 or 80 mM in high-glucose-induced HK-2 cells	Anti-inflammatory	• Inflammatory response (TLR4, MyD88, p-IRAK4, p-IKKβ, and p-NF-κBp65) ↓	
	10, 20, 40, and 80 µg/mL in phytohemagglutinin-induced human peripheral blood mononuclear cells	Immunomodulatory and anti-inflammatory	• Inflammation (TNF-α, IL-6, and IL-8) ↓ • Anti-inflammation (IL-10) ↑	[60]
	Non-cell-based in vitro assay	Antidiabetic and antioxidant	• DPPH (IC_50_ = 32.7 and 109.5 µg/mL µg/mL) and ABTS (IC_50_ = 37.5 and 151.5 µg/mL), superoxide ion and radical scavenging activity • ORAC activity (IC_50_ = 25.6 µg/mL) • Fe^3+^ reducing activity • Linoleic acid peroxidation inhibitory activity (IC_50_ = 25.6 µg/mL) • α-Amylase inhibitory (IC_50_ = 31.9 µg/mL) activity • Antiglycation activity (IC_50_ = 79.1 µg/mL)	[31,53,60,61]
	3.75–60 µg/mL in L6-myotubes and FeSO4-induced Chang cells		• Glucose uptake in in L6-myotubes (EC_50_ = 112 µg/mL) ↑ • LPO in Chang cells (IC_50_ = 59.0 µg/mL) ↓	
Geraniin	Non-cell-based in vitro assay	Antioxidant, anti-inflammatory, and antihypertensive	• DPPH (IC_50_ = 0.92 µM), hydroxyl (IC_50_ = 0.11 µM), and superoxide (IC_50_ = 2.56 µM) radicals scavenging activity • SSAO inhibitory activity (IC_50_ = 0.70 µM) • ACE inhibitory activity (IC_50_ = 13.22 and 400 µM; 55% inhibition at 0.0001 µM)	[44,47,49]
	0.25 mM in ethanol-induced rat liver slices	Antioxidant, antiglycation, and hepatoprotective	• Apoptosis (Bax:Bcl-2 ratio and LDH release) ↓ • LPO ↓ • PCC ↓ • CAT, SOD, GPx, and GR ↑ • Oxidative DNA damage (8-OHdG) ↓	[38]
Chebulinic acid	Non-cell-based in vitro assay	Antioxidant	• DPPH (IC_50_ = 12.5 and 4.5 µM), ABTS (IC_50_ = 3.5 µM) and NO (IC_50_ = 54.6 µM) radical scavenging activity • Fe^3+^ reducing activity (IC_50_ = 22.5 µM)	[43,62]
	50 µM in erastin-treated bone marrow-derived mesenchymal stem cells		• ROS production ↓	
	26 µM in tBHP-induced L-02 human hepatocytes	Antioxidant, anti-inflammatory and hepatoprotective	• ROS production ↓ • MAPK signaling (p-ERK, JNK and p38 MAPK) ↑ • Nrf2/HO-1 signaling ↑ • Cellular LDH leakage ↓	[43]
	5–100 µM in LPS-stimulated RAW 264.7 cells	Anti-inflammatory	• NO production (IC_50_ = 53.4µM) ↓ • TNF-α, IL-6, iNOS, and COX-2 ↓	[42]
Chebulagic acid	Non-cell-based in vitro assay	Antidiabetic	• α-Glycosidase inhibitory (IC_50_ = 37 µg/mL)	[40]
	0.05–0.5 mM in maltose-treated Caco-2 cells		• α-Glycosidase activity and maltose hydrolysis ↓ • Glucose uptake ↑	
	10, 50, 100 µM in 3T3-L1 pre-adipocyte	Antidiabetic, anti-dyslipidemic, and anti-obesogenic	• Pre-adipocytes differentiation (PPARγ and C/EBPα expression; adiponectin levels) ↑ • Insulin signaling (GLUT4) ↑ • Glucose uptake ↑	[41]
Corilagin	Non-cell-based in vitro assay	Antihypertensive	• ACE inhibitory activity (IC_50_ = 3.7 mM)	[44]
Punicalin	Non-cell-based in vitro assay	Antioxidant	• DPPH (IC_50_ = 0.024 mM), hydroxyl (IC_50_ = 0.061 mM), and superoxide radical scavenging activity • Fe_3+_ reducing activity • LPO inhibitory activity	[53,55]
	10, 20, 40, and 80 µg/mL in phytohemagglutinin-induced human peripheral blood mononuclear cells	Immunomodulatory and anti-inflammatory	• Inflammation (TNF-α, IL-6, and IL-8) ↓ • Anti-inflammation (IL-10) ↑	[60]
Amariin	0.25 mM in ethanol-induced rat liver slices	Antioxidant, antiglycation, and hepatoprotective	• Apoptosis (Bax:Bcl-2 ratio and LDH release) ↓ • LPO ↓ • PCC ↓ • CAT, SOD, GPx, and GR ↑ • Oxidative DNA damage (8-OHdG) ↓	[38]
Vescalagin	Non-cell-based in vitro assay	Antidiabetic and antioxidant	• DPPH (IC_50_ = 9.65 µg/mL), ABTS (IC_50_ = 4.90 µg/mL) and DMPD (IC_50_ = 8.73 µg/mL) radical scavenging activity • Fe^3+^ and Cu^2+^ reducing activity • α-Glycosidase inhibitory activity IC_50_ = 13.15 µg/mL)	[56]
	2 and 5 mg/mL in STC1 cells	Antidiabetic	• Insulin stimulatory potential (GLP1 secretion) ↑	[37]
Pedunculagin, Castalagin, Sanguiin H-6, Acutissimin A, and Casuarictin	2 and 5 mg/mL in STC1 cells	Antidiabetic	• Insulin stimulatory potential (GLP1 secretion) ↑	[37]
Grandinin	Non-cell-based in vitro assay	Antioxidant	• DPPH scavenging activity (IC_50_ = 4.3 µg/mL)	[51]

Abbreviations: ‘↑’, increase or up-regulation; ‘↓’, decrease or down-regulation; ‘8-OHdG’, 8-hydroxy-2′-deoxyguanosine; ‘ABTS’, 2,2′-azino-bis-(3-ethylbenzothiazoline-6-sulfonic acid); ‘ACE’, angiotensin-converting enzyme; ‘Akt’, protein kinase B; ‘Bax’, Bcl-2-associated X protein; ‘Bcl-2’, B-cell lymphoma 2; ‘C/EBPα’, CCAAT-enhancer binding protein alpha; ‘CAT’, catalase; ‘COX-2’, cyclooxygenase 2; ‘DMPD’, N,N-dimethyl-p-phenylenediamine dihydrochloride; ‘DPPH’, 2,2-diphenyl-1-picrylhydrazyl; ‘ERK’, extracellular signal-regulated kinase; ‘GLP1’, glucagon-like peptide; ‘GLUT4’, glucose transporter type 4; ‘GPx’, glutathione peroxidase; ‘GR’, glutathione reductase; ‘GS’, glycogen synthase; ‘GSK3β’, glycogen synthase kinase-3; ‘HO-1’, heme oxygenase-1; ‘IKKβ’, inhibitor of nuclear factor kappa-B kinase subunit beta; ‘IRAK4’, interleukin-1 receptor-associated kinase 4; ‘IL’, interleukin; ‘iNOS’, inducible nitric oxide synthase; ‘JNK’, c-Jun N-terminal kinase; ‘LDH’, lactate dehydrogenase; ‘LPO’, lipid peroxidation; ‘LPS’, lipopolysaccharide; ‘MAPK’, mitogen-activated protein kinase; ‘MyD88’, myeloid differentiation primary response 88; ‘NF-κB p65’, nuclear factor kappa-light-chain-enhancer of activation B cells (transcription factor 65); ‘NO’, nitric oxide; ‘Nrf2’, nuclear factor erythroid 2-related factor 2; ‘ORAC’, oxygen radical absorbance capacity; ‘PCC’, protein carbonyl content; ‘PEPCK’, phosphoenolpyruvate carboxykinase; ‘PI3K’, phoshoinositide 3-kinase; ‘PPARγ’, peroxisome proliferator-activated receptor gamma; ‘ROS’, reactive oxygen species; ‘SOD’, superoxide dismutase; ‘SSAO’, semi carbazide-sensitive amine oxidase; ‘tBHP’, tertiary butyl hydroperoxide; ‘TNF-α’, tumor necrosis factor alpha; ‘TLR4’, toll-like receptor 4.

**Table 3:** In vivo or pre-clinical pharmacological Potential of Ellagitannins in Disorders of Metabolic Syndrome and Associated Inflammatory Responses.

Ellagitannins	Reported Dose	Animal Model	Medicinal Potential	Effect/Molecular Targets	References
Punicalagin	100, 150, and 200 mg/kg (oral; 4 wk)	HFD/STZ-induced C57BL/6J mice	Antidiabetic, anti-dyslipidemic, anti-inflammatory, and tissue-protective.	• Glycogenesis p-GS and p-GSK3β) ↑ • TG and TC ↓ • Gluconeogenesis (PEPCK) ↓ • Insulin signaling (PI3K and p-Akt) ↑ • Hepatic, pancreatic and renal damage ↓ • Tissue inflammation (IL-6, TNF-α, p-IRAK4, p-IKKβ, p-IκBα, p-NF-κBp65, MCP-1, HMGB1, MyD88, and TLR4) ↓	[54]
	0.5–15 mg/kg (i.v.; single)	SHRs	Antihypertensive	• ABP ↓	[39]
	10 mg/kg (oral; 21 d)	Oxidized fish oil-fed BALB/c mice	Antioxidant, anti-inflammatory, and tissue-protective	• Systemic, hepatic and intestinal SOD and GPx ↑ • Systemic and tissue LPO ↓ • Intestinal inflammation (TNF-α and IL-6) ↓ • Tissue damage ↓	[53]
Geraniin	5 and 10 mg/kg; 10 and 50 mg/kg; 3.125–100 mg/kg (oral; 4 wk)	HFD-fed C57BL/6 mice and SD rats	Antidiabetic, antihypertensive; anti-obesogenic, anti-dyslipidemic, antioxidant, anti-inflammatory, and antiglycation	• Glucose tolerance ↑ • HOMA-IR ↓ • TG, LDL-c, and TC↓ • HDL-c ↑ • Insulin signaling and fatty acid oxidation (PPARα and adiponectin) ↑ • Tissue inflammation (IL-1β) ↓ • LPO, PCC, and AGEs ↓ • SOD, GPx, GR, and GSH ↑ • SBP and renin concentration ↓	[28,48,50]
	5 mg/kg (oral; single)	SHRs	antihypertensive	• SBP and DBP ↓	[47]
	3 and 30 mg/kg (i.p.; single)	Acetic acid-induced mice	Antinociceptive and tissue-protective	• No. of abdominal constrictions ↓	[46]
Corilagin	5 and 10 mg/kg (oral; 15 wk)	HFD-fed C57BL/6 mice	Antidiabetic, anti-dyslipidemic, antioxidant, and hepatoprotective	• Glucose and insulin tolerance ↑ • HOMA-IR ↓ • TG, LDL-c, and TC↓ • HDL-c ↑ ↑ • Lipolysis (FFA and FABP4) ↓ • Hepatic damage (ALT, AST, and NAFLD score) ↓	[57]
	0.5–20 mg/kg (i.v.; single)	SHRs	Antihypertensive	• ABP ↓	[39]
Chebulinic acid	37.5, 75 and 150 mg/kg (oral; 5 d)	CCl_4_-induced mice and ACP-induced zebrafish	Hepatoprotective, antioxidant and anti-inflammatory	• SOD activity ↑ • LPO ↓ • Hepatic damage (ALT and AST) ↓ • Nrf2/HO-1 ↑	[43]
	0.5–10 mg/kg (i.v.; single)	SHRs	Antihypertensive	• ABP ↓	[39]
Chebulagic acid	100 mg/kg (oral; single)	Normal SD rats	Anti-hyperglycemic	• Glucose tolerance ↑	[40]
	35 mg/kg (i.p.; 4 wk)	HFD/STZ-induced Wistar rats	Antidiabetic, antioxidant, anti-inflammatory, and tissue-protective	• HOMA-IR ↓ • Insulin signaling (GLUT4, IR, IRS-1, and Akt) ↑ • Renal dysfunction (creatinine and urea) ↓ • Inflammation (IL-1β, IL-6, TNF-α & NF-kB p65) ↓ • Anti-inflammation (IL-10) ↑ • LPO ↓ • GSH, SOD, CAT, GPx, and GR ↑	[63]
Punicalin	10 mg/kg (oral; 21 d)	Oxidized fish oil-fed BALB/c mice	Antioxidant, anti-inflammatory, and tissue-protective	• Systemic, hepatic and intestinal SOD and GPx ↑ • Systemic and tissue LPO ↓ • Intestinal inflammation (TNF-α and IL-6) • Tissue damage ↓	[53]
Grandinin	35 and 70 mg/kg (i.p.; 7 d)	Normal and STZ-induced mice	Antidiabetic, antioxidant, and tissue-protective	• Glucose tolerance ↑ • Renal dysfunction (urea) ↓ • LPO ↓	[51]
Castalagin	0.5–20 mg/kg (i.v.; single)	SHRs	Antihypertensive	• ABP ↓	[39]
Sanguiin H-6	100 mg/kg (oral; single)	Normal SD rats	Anti-hyperglycemic	• Glucose tolerance ↑ • Disaccharidase activity ↓	[40]
Furosin	3 and 30 mg/kg (i.p.; single)	Acetic acid-induced mice	Antinociceptive and tissue-protective	• No. of abdominal constrictions ↓	[46]
Davidiin	90 mg/kg (4 wk)	HFD/STZ-induced SD rats	Antidiabetic	• Blood glucose ↓	[45]
Oenothein B	640-µM treatment	Normal and 10 mM glucose-treated *C. elegans*	Antioxidant and anti-obesogenic	• ROS production ↓ • SOD and GPx ↑ • Fat accumulation ↓ • TG ↓ • mRNA of key genes involved in lipogenesis and fatty acid desaturation ↓	[52]

Abbreviations: ‘↑’, increase or up-regulation; ‘↓’, decrease or down-regulation; ‘ABP’, arterial blood pressure; ‘ACP’, acetaminophen; ‘AGEs’, advanced glycation end products; ‘Akt’, protein kinase B; ‘ALT’, alanine transaminase; ‘AST’, aspartate aminotransferase; ‘CAT’, catalase; ‘CCl4’, carbon tetrachloride; ‘DBP’, diastolic blood pressure; ‘FABP4’, fatty acid-binding protein 4; ‘FFA’, free fatty acid; ‘GLUT4’, glucose transporter type 4; ‘GPx’, glutathione peroxidase; ‘GSH’, reduced glutathione; ‘GR’, glutathione reductase; ‘GS’, glycogen synthase; ‘GSK3β’, glycogen synthase kinase-3; ‘HDL-c’, high-density lipoprotein cholesterol; ‘HFD’, high-fat diet; ‘HMGB1’, high-mobility group protein 1; ‘HO-1’, heme oxygenase-1; ‘HOMA-IR’, Homeostatic Model Assessment for Insulin Resistance; ‘IκBα’, nuclear factor of kappa light polypeptide gene enhancer in B-cells inhibitor alpha; ‘IKKβ’, inhibitor of nuclear factor kappa-B kinase subunit beta; ‘IRAK4’, interleukin-1 receptor-associated kinase 4; ‘IL’, interleukin; ‘i.p.’, intraperitoneal; ‘IRS-1’, insulin receptor substrate 1; ‘i.v.’; intravenous; ‘LDL-c’, low-density lipoprotein cholesterol; ‘LPO’, lipid peroxidation; ‘MCP-1’, monocyte chemoattractant protein-1; ‘MyD88’, myeloid differentiation primary response 88; ‘NAFLD’, non-alcoholic fatty acid liver disease; ‘NF-kB p65’, nuclear factor kappa-light-chain-enhancer of activation B cells (transcription factor 65); ‘Nrf2’, nuclear factor erythroid 2-related factor 2; ‘PCC’, protein carbonyl content; ‘PEPCK’, phosphoenolpyruvate carboxykinase; ‘PI3K’, phoshoinositide 3-kinase; ‘PPARα’, peroxisome proliferator-activated receptor alpha; ‘ROS’, reactive oxygen species; ‘SBP’, systolic blood pressure; ‘SD’, Sprague–Dawley; ‘SHRs’, spontaneously hypertensive rats; ‘SOD’, superoxide dismutase; ‘STZ’, streptozotocin; ‘TC’, total cholesterol; ‘TG’, triglycerides; ‘TNF-α’, tumor necrosis factor alpha; ‘TLR4’, toll-like receptor 4.

## Data Availability

No new data were created or analyzed in this study. Data sharing is not applicable to this article.

## Abbreviations

ABTS	2,2′-azino-bis-3-ethylbenzothiazoline-6-sulfonic acid
ACE	angiotensin-converting enzyme
AGEs	advanced glycation end products
Akt	protein kinase B
ALT	alanine transaminase
AST	aspartate aminotransferase
C/EBP	CCAAT-enhancer binding protein
CAT	catalase
COX-2	cyclooxygenase 2
CVD	cardiovascular disease
DPPH	2,2-diphenyl-1-picrylhydrazyl
GLP-1	glucagon-like peptide
GLUT4	glucose transporter type 4
GPx	glutathione peroxidase
GSH	reduced glutathione
GR	glutathione reductase
HDL-C	high-density lipoprotein cholesterol
HFD	high-fat diet
HHDP	hexahydroxydiphenoyl
HOMA-IR	homeostatic model assessment for insulin resistance
IL	interleukin
iNOS	inducible nitric oxide synthase
IRS-1	insulin receptor substrate 1
LDH	lactate dehydrogenase
LDL-C	low-density lipoprotein cholesterol
MASLD	metabolic dysfunction-associated steatotic liver disease
MCP-1	monocyte chemoattractant protein-1
MDA	malondialdehyde
MetS	metabolic syndrome
NAFLD	non-alcoholic fatty acid liver disease
NF-kB p65	nuclear factor kappa-light-chain-enhancer of activation B cells (transcription factor 65)
NO	nitric oxide
ORAC	oxygen radical absorbance capacity
PCC	protein carbonyl content
PI3K	phoshoinositide 3-kinase
PPARy	peroxisome proliferator-activated receptor gamma
ROS	reactive oxygen species
SD	Sprague–Dawley
SHRs	spontaneously hypertensive rats
SOD	superoxide dismutase
SREBP-1	sterol regulatory element-binding protein
STZ	streptozotocin
T2D	type 2 diabetes
tBHP	tertiary butyl hydroperoxide
TC	total cholesterol
TG	triglycerides
TNF-α	tumor necrosis factor alpha
